# The guidance ratio: a measure of post-diagnostic antibiotic decision-making

**DOI:** 10.1093/jacamr/dlag195

**Published:** 2026-09-01

**Authors:** Kalle Garpvall, Alex Aljundi, Anna Dahl, Ellinor Sterky, Joachim Luthander, Susanne Sütterlin

**Affiliations:** Astrid Lindgren Children's Hospital, Karolinska University Hospital, Stockholm, Sweden; Department of Women's and Children's Health, Pediatric Nephrology and Infectious Diseases (PNID) Research Group, Uppsala University, Uppsala, Sweden; Department of Women's and Children's Health, Division of Clinical Paediatrics, Karolinska Institutet, Stockholm, Sweden; Astrid Lindgren Children's Hospital, Karolinska University Hospital, Stockholm, Sweden; Astrid Lindgren Children's Hospital, Karolinska University Hospital, Stockholm, Sweden; Department of Women's and Children's Health, Division of Clinical Paediatrics, Karolinska Institutet, Stockholm, Sweden; Department of Women's and Children's Health, Pediatric Nephrology and Infectious Diseases (PNID) Research Group, Uppsala University, Uppsala, Sweden; Department of Pediatrics, Uppsala University Hospital, Uppsala, Sweden

## Abstract

**Background:**

Scalable measures that capture post-diagnostic antibiotic decisions are lacking. We developed and validated the guidance ratio (GR), a registry-based measure of post-diagnostic antibiotic decision-making.

**Methods:**

We conducted a retrospective multi-centre validation study of the GR and discontinuation ratio (DR), two registry-based indicators of post-diagnostic antibiotic decision-making in paediatric febrile urinary tract infection. Prescribing outcomes were classified using rule-based algorithms and compared with manually classified outcomes from medical record review. Indicator performance was evaluated against medical record data used as the reference standard.

**Results:**

Among 909 episodes, the GR identified guided treatment with a sensitivity of 0.78 and specificity of 1.00. Although registry-based estimates underestimated absolute values, they closely tracked temporal prescribing patterns. Calibration substantially improved agreement with manually classified outcomes.

**Conclusions:**

The GR captured clinically meaningful prescribing patterns and may support scalable monitoring of post-diagnostic antibiotic decision-making in stewardship programmes.

## Graphical Abstract

**Graphical Abstract:**
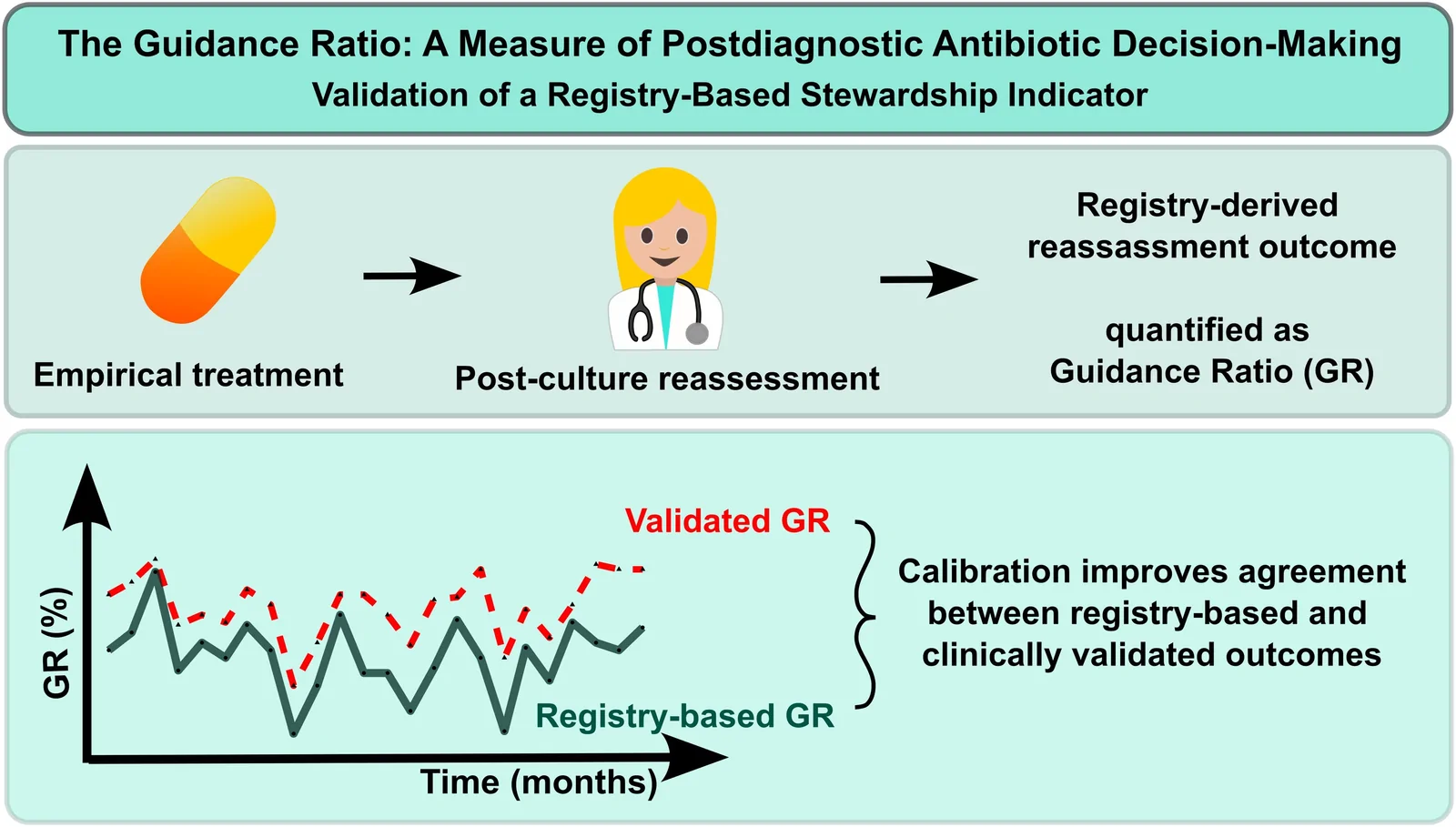

## Introduction

Antimicrobial stewardship relies on quality indicators to monitor antibiotic use, yet most existing measures focus on antibiotic exposure, such as prescription rates, treatment duration, and choice of agent. While these indicators are useful for surveillance, they do not capture the quality of clinical decision-making, particularly decisions made after diagnostic reassessment.^1,2^ In many clinical scenarios, including paediatric febrile urinary tract infection (UTI), empirical antibiotic treatment is initiated under diagnostic uncertainty. When microbiological and clinical information becomes available, clinicians reassess the diagnosis and decide whether to continue, modify, or discontinue treatment.^3^ This post-diagnostic decision point is central to antimicrobial stewardship but is rarely reflected in existing indicators.^4^

Electronic prescribing registries provide access to longitudinal clinical and microbiological data and may enable the development of scalable indicators that capture these decisions. We propose two registry-based indicators, the guidance ratio (GR) and discontinuation ratio (DR), to quantify antibiotic decision-making after diagnostic reassessment. Rule-based classifications derived from registry data require validation against clinically verified outcomes to ensure that they reflect true clinical reasoning. Paediatric febrile UTI represents a useful model condition because treatment is commonly initiated empirically and subsequently reassessed when microbiological results become available, creating a well-defined post-diagnostic decision point.

In this study, we evaluate the validity of GR and DR against manually classified outcomes and assess systematic differences between registry-based and clinically verified measures.

## Material and methods

### Study design and setting

We conducted a retrospective multi-centre validation study at three Swedish university paediatric emergency departments (University Children’s Hospitals of Uppsala, and Solna and Huddinge in Stockholm). All children aged 0–18 years treated empirically for suspected febrile UTI during the study period were eligible. The study was approved by the Swedish Ethical Review Board (document ID 2020-03297), informed consent was not required.

### Data source and case identification

Data were retrieved from the Swedish antibiotic prescribing registry (‘Infektionsverktyget’), which links antibiotic prescriptions to electronic medical records. Eligible patients were identified using this registry. At all participating centres, the prescribing clinician was required to register the indication for each antibiotic prescription. All antibiotic treatment episodes registered with the indication febrile UTI during the study period were identified and included. Episodes of febrile UTI were identified and linked to both initial empirical treatment and subsequent prescribing decisions. The study period extended from 1 September 2021 to 31 August 2023 in Uppsala, from 9 December 2022 to 31 May 2023 in Solna, and from 9 December 2022 to 9 June 2023 in Huddinge. All included episodes underwent medical record review. In the Uppsala dataset, detailed follow-up documentation enabled manually classified classification of prescribing outcomes.

### Definitions and indicators

Empirical treatment was defined as antibiotic therapy initiated before microbiological results were available. Following diagnostic reassessment, episodes were classified as either guided treatment or discontinuation. Guided treatment was defined as modification of empirical antibiotic therapy. Discontinuation was defined as cessation of antibiotic therapy usually when UTI was considered unlikely. During the study period, Swedish treatment recommendations favoured empirical oral third-generation cephalosporins (cefixime/ceftibuten, available on licence), followed by de-escalation to a narrower-spectrum agent whenever supported by culture and susceptibility results. Consequently, confirmed febrile UTI generally represented an expected guided treatment episode.

Data were exported from Infektionsverktyget. Antibiotic prescriptions were sorted chronologically for each patient. The first prescription in each treatment episode represented empirical therapy. Subsequent prescriptions represented post-diagnostic treatment. Applying pre-defined rules to the prescription sequence yielded the registry-based GR and DR.

In the validation dataset, prescribing outcomes were determined by clinician review of follow-up documentation and microbiological findings. For the manually classified reference standard, the final diagnosis assigned by the follow-up clinician after review of the clinical course, urine culture and antimicrobial susceptibility results was used to determine the expected stewardship outcome. Cases with a confirmed febrile UTI were classified as guided treatment, whereas cases in which febrile UTI was not confirmed were classified as discontinuation. This reference standard assumes that the post-diagnostic clinical assessment reflects the stewardship decision. Validation against the manually reconstructed reference standard was performed in the Uppsala cohort only, as complete reference data were available only from this centre.

The primary indicator was the GR, defined as the proportion of empirically treated episodes that resulted in guided treatment after diagnostic reassessment. To quantify post-diagnostic prescribing decisions, the two indicators were calculated:


$$\mathrm{Guidance}\;\mathrm{ratio}\;(\mathrm{GR})=\frac{\mathrm{number}\;\mathrm{of}\;\mathrm{guided}\;\mathrm{treatments}}{\mathrm{number}\;\mathrm{of}\;\mathrm{empirical}\;\mathrm{treatments}}$$

$$\begin{array}{r} \mathrm{Discontinuation}\;\mathrm{ratio}\;(\mathrm{DR})=\frac{\mathrm{number}\;\mathrm{of}\;\mathrm{discontinued}\;\mathrm{treatments}}{\mathrm{number}\;\mathrm{of}\;\mathrm{empirical}\;\mathrm{treatments}} \end{array}$$

### Statistical analysis

The performance of registry-based classifications was evaluated against the manually classified reference standard using sensitivity and specificity. Agreement between registry-based and clinically validated GR was assessed using Bland–Altman analysis and Lin’s concordance correlation coefficient.^5^ A calibration model was constructed to quantify and correct systematic differences between registry-based and clinically validated measures. Monthly values of GR and DR were summarized using percentile intervals to define expected variation over time. Data were compiled and pre-processed in Microsoft Excel (Microsoft Corp., Redmond, WA, USA) and subsequently analysed and visualized in R version 4.5.1 (R Foundation for Statistical Computing, Vienna, Austria).

### Exploratory analysis

An exploratory diagnostic evidence score was constructed to assess the relationship between diagnostic certainty and prescribing decisions. One point was assigned for each of five pre-defined clinical or microbiological criteria: body temperature >38.5°C, leukocyte esterase ≥2+, positive nitrite, C-reactive protein > 70 mg/L, and significant growth of a relevant uropathogen in urine culture.^6^ The resulting score (0–5) reflected the level of diagnostic support for febrile UTI at reassessment. Missing values were assigned zero points.

## Results

### Study population

A total of 909 febrile UTI episodes were included across all study sites (Uppsala n = 431, Solna n = 336, and Huddinge n = 142). The median age was 2 years (IQR 0.7–5), and 84% of patients were girls. Baseline clinical characteristics and prescribing patterns are summarized in Table 1.

**Table 1.** Baseline characteristics and prescribing patterns across study sites

Variable	Huddinge	Uppsala	Solna	Total
Total	142	431	336	909
Age (years)	2 (0.7–4)	2 (0.8–5)	1 (0.6–5)	2 (0.7–5)
Sex (female)	114/142 (80%)	358/431 (83%)	289/336 (86%)	768/909 (84%)
Urine culture significant	57/125 (46%)	215/417 (51%)	154/319 (48%)	426/861 (49%)
Empirical treatment				
Cefixim/ceftibuten	112/142 (78%)	394/431 (91%)	299/336 (89%)	805/909 (89%)
Other	24/142 (17%)	23/431 (5%)	23/336 (6%)	70/909 (8%)
None	6/142 (4%)	14/431 (3%)	14/336 (4%)	34/909 (4%)
Guided treatment				
Cefixim/ceftibuten	22/142 (16%)	47/431 (12%)	38/336 (11%)	113/909 (13%)
Co-trimoxazole	56/142 (39%)	164/431 (38%)	101/336 (30%)	321/909 (35%)
Amoxicillin clavulanic acid	7/142 (5%)	6/431 (1%)	19/336 (6%)	32/909 (4%)
Ciprofloxacin	2/142 (1%)	17/431 (4%)	12/336 (4%)	31/909 (3%)
Other	7/142 (5%)	36/431 (8%)	19/336 (6%)	62/909 (7%)
None	47/142 (33%)	156/431 (36%)	147/336 (44%)	350/909 (39%)

Data from paediatric patients with febrile UTI at three university-affiliated emergency departments in Sweden. Continuous variables are presented as median (IQR) and categorical variables as n (%)

### Validation of prescribing outcomes

In the Uppsala validation dataset (n = 431), manual review classified 273/431 episodes (63%) as guided treatment, while 119/431 (28%) were discontinued and 39/431 (9%) lacked sufficient follow-up documentation. The registry-based classification identified 219/431 (51%) episodes as guided treatment and 156/431 (36%) as discontinuation. Compared with the manually classified reference standard, the registry-based classification demonstrated a sensitivity of 0.78 and a specificity of 1.00 for identifying guided treatment.

### Systematic differences between registry-based and validated measures

**Figure 1.**
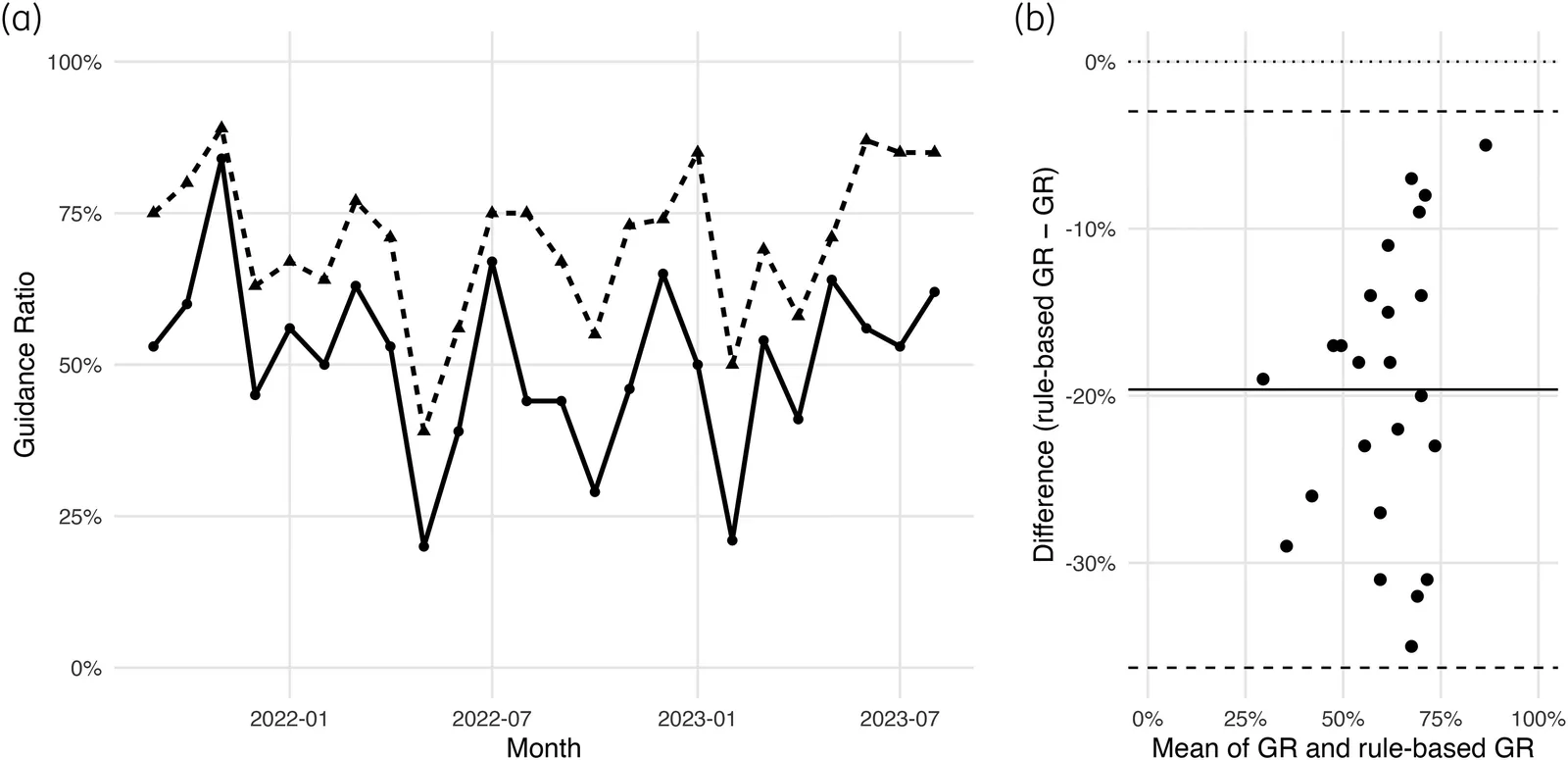
Comparison of validated and registry-based GR in the Uppsala dataset. (a) Monthly values of GR based on manually classified outcomes (dashed line) and rule-based classification (solid line). (b) Bland–Altman plot showing the difference between validated and rule-based GR (rule-based GR—validated GR) plotted against their mean values. The solid line represents the mean difference (bias), and dashed lines indicate limits of agreement (±1.96 SD).

The registry-based GR consistently underestimated the clinically validated GR but accurately captured temporal prescribing patterns. Across the study period, the mean absolute error between measures was 0.20, with a negative bias indicating systematic underestimation by the registry-based classification. Agreement between absolute GR estimates was modest (Lin’s concordance correlation coefficient of 0.39) (Figure 1).

To account for this systematic bias, a linear regression model was fitted with validated GR as the dependent variable. This yielded the calibration function:


ValidatedGR≈0.349+0.699×(rule−basedGR).

Application of this function reduced discrepancies and resulted in close agreement with the validated GR across the full study period (Figure 1).

### Secondary analyses

**Figure 2.**
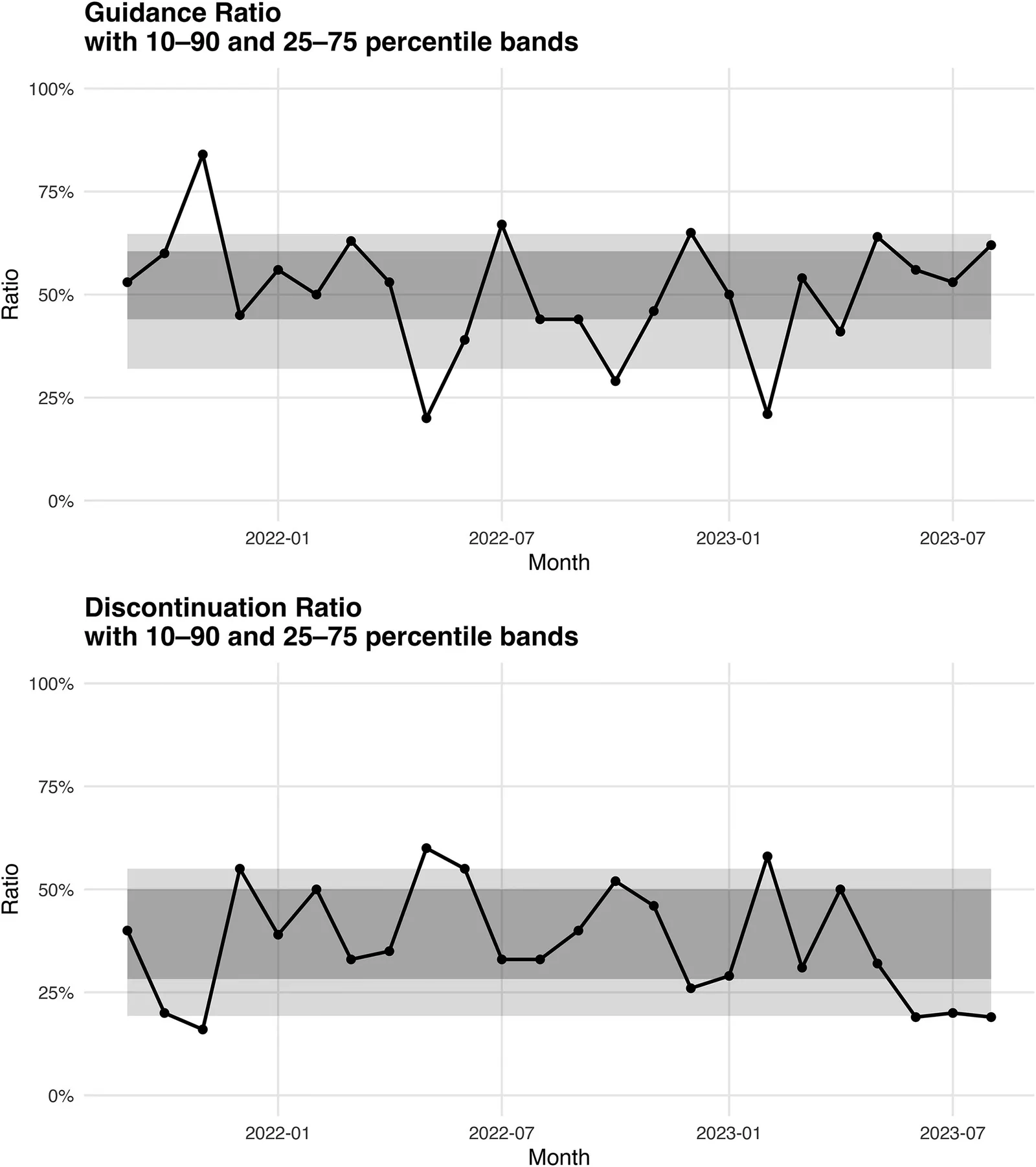
Monthly trends in registry-based prescribing indicators in the Uppsala dataset. GR and DR calculated per month for empirically treated episodes of suspected febrile UTI. Shaded areas represent percentile intervals, with the darker band indicating the interquartile range and the lighter band the 10th–90th percentile range.

The DR showed an inverse pattern to GR and ranged from 16% to 60% across months. Most values fell within expected percentile ranges, with occasional deviations but no consistent temporal trend (Figure 2).

Across study sites, the overall registry-based GR ranged from 45% to 51%, with a pooled value of 49%, while treatment discontinuation ranged from 33% to 44%. When stratified by diagnostic evidence, GR increased consistently with a higher number of fulfilled criteria, supporting the construct validity of the indicators (Table 2).

**Table 2.** Registry-based GR stratified by diagnostic evidence across study sites

Diagnostic criteria	Huddinge	Uppsala	Solna
0	7/13 (54%)	2/24 (8%)	4/23 (17%)
1	15/39 (38%)	21/84 (25%)	18/98 (18%)
2	21/45 (47%)	54/130 (42%)	45/101 (45%)
3	15/19 (79%)	71/107 (66%)	39/61 (64%)
4	11/18 (61%)	45/55 (82%)	34/38 (89%)
5	6/8 (75%)	26/31 (84%)	13/15 (87%)
Overall GR	75/142 (46%)	219/431 (51%)	153/336 (45%)

Diagnostic evidence score (0–5) based on clinical and microbiological criteria as defined in Methods.

## Discussion

The main finding of this study is that the GR can serve as a scalable measure of post-diagnostic antibiotic decision-making. In a multi-centre cohort of paediatric febrile UTI, registry-based GR demonstrated high specificity and moderate sensitivity for identifying manually classified guided treatment. Although registry-based estimates systematically underestimated clinically validated GR, they accurately captured temporal prescribing patterns and could be calibrated to improve agreement. These findings suggest that GR may provide a practical stewardship metric in settings where large-scale clinical review is not feasible.

Previous stewardship evaluations have highlighted the limitations of exposure-based metrics for assessing prescribing quality, as consumption measures cannot account for clinical context or appropriateness of therapy.^7–10^ In addition, systematic reviews of stewardship indicators have shown that validated outcome measures remain rare and that testing against clinically meaningful outcomes is needed.^7,10^ In line with these observations, our findings show that GR captures aspects of post-diagnostic clinical decision-making that are not reflected in conventional exposure-based measures. Across the three study sites, registry-based GR ranged from 45% to 51%, while treatment discontinuation ranged from 33% to 44%. The registry-based classification demonstrated high specificity (1.00) and moderate sensitivity (0.78) for identifying guided treatment and accurately captured temporal prescribing patterns despite systematic underestimation of absolute values. The observed specificity of 1.00 reflects the conservative nature of the registry-based classification. The algorithm only classified treatment as guided when the prescription sequence clearly indicated a post-diagnostic treatment decision, thereby minimizing false-positive classifications at the expense of some loss in sensitivity. Similar discrepancies between registry-based stewardship indicators and manually classified outcomes have been reported in previous validation studies using electronic health record data.^8,9,11^ Importantly, agreement improved substantially after calibration, indicating that systematic bias in registry-based stewardship measures can be addressed using established approaches for method comparison and measurement error.^12,13^

The stepwise increase in GR with stronger diagnostic evidence provides additional support for its construct validity. Across all study sites, GR increased consistently with the number of fulfilled clinical and microbiological criteria, indicating that prescribing behaviour followed clinically expected patterns across diagnostic strata. Lower diagnostic certainty was associated with lower GR values and higher discontinuation rates, consistent with appropriate reassessment and discontinuation of empirical therapy when infection was considered unlikely. Similar associations between diagnostic certainty and antibiotic continuation have been described in paediatric febrile UTI and other empirically treated infections, where post-diagnostic reassessment represents a key stewardship opportunity.^3,4^

The observed month-to-month variation in GR further illustrates how registry-based indicators may be used to monitor prescribing behaviour over time. Although absolute values were systematically underestimated, registry-based GR captured temporal prescribing patterns and identified periods that deviated from expected variation, providing a practical starting point for stewardship review. More advanced approaches, including structured electronic documentation and automated analysis of clinical free text, may further improve the ability of registry-based measures to capture clinical reasoning and reduce systematic misclassification.^2,8,9^

An important limitation of registry-based stewardship measures is their inability to fully capture the clinical reasoning underlying treatment decisions. Previous studies have similarly reported discrepancies between algorithm-based classifications and manually classified outcomes, particularly when follow-up documentation is incomplete or when relevant contextual information is unavailable.^9,10^ In our study, registry-based GR captured overall prescribing patterns well but systematically underestimated clinically validated GR. GR should therefore be viewed as a scalable proxy measure of post-diagnostic antibiotic decision-making rather than a direct measure of prescribing appropriateness. This finding reinforces the importance of validation against manually classified outcomes and supports the use of calibrated rather than unadjusted registry estimates when interpreting stewardship performance. International stewardship initiatives, including the DRIVE-AB programme, have likewise emphasized that validated outcome measures remain rare despite their importance for assessing prescribing quality.^14^

In conclusion, the GR captures clinically meaningful post-diagnostic antibiotic decision-making and represents a stewardship outcome measure that can be derived from routinely collected registry data. While validation and calibration remain necessary for accurate interpretation, GR offers a scalable approach for monitoring antibiotic reassessment beyond traditional exposure-based metrics. The framework may be applicable to other empirically treated infections where microbiological findings inform post-diagnostic antibiotic decisions and to healthcare systems with linked prescribing and microbiological data.
